# Same same-but different: using qualitative studies to inform concept elicitation for quality of life assessment in telemedical care: a request for an extended working model

**DOI:** 10.1186/s12955-021-01807-8

**Published:** 2021-07-05

**Authors:** Klara Greffin, Silke Schmidt, Neeltje van den Berg, Wolfgang Hoffmann, Oliver Ritter, Michael Oeff, Georg Schomerus, Holger Muehlan

**Affiliations:** 1 Department of Psychology, Chair of Health and Prevention, University of Greifswald, Robert-Blum-Str. 13, 17489 Greifswald, Germany; 2 Institute of Community Medicine, Section Epidemiology of Health Care and Community Health, University Medicine Greifswald, Ellernholzstraße 1-2, 17475 Greifswald, Germany; 3 Brandenburg City Hospital, Brandenburg Medical School Theodor Fontane, Fehrbelliner Str. 38, 16816 Neuruppin, Germany; 4 Brandenburg City Hospital, Hochstraße 29, 14770 Brandenburg an der Havel, Germany; 5 Department of Psychiatry and Psychotherapy, University Medicine Leipzig, Semmelweisstraße 10, 04103 Leipzig, Germany

**Keywords:** Telemedicine, Quality of life, Patient-reported outcome measure, Concept elicitation

## Abstract

**Background:**

Although telemedical applications are increasingly used in the area of both mental and physical illness, there is no quality of life (QoL) instrument that takes into account the specific context of the healthcare setting. Therefore, the aim of this study was to determine a concept of quality of life in telemedical care to inform the development of a setting-sensitive patient-reported outcome measure.

**Methods:**

Overall, 63 semi-structured single interviews and 15 focus groups with 68 participants have been conducted to determine the impact of telemedical care on QoL. Participants were patients with chronic physical or mental illnesses, with or without telemedicine supported healthcare as well as telemedical professionals. Mayring's content analysis approach was used to encode the qualitative data using MAXQDA software.

**Results:**

The majority of aspects that influence the QoL of patients dealing with chronic conditions or mental illnesses could be assigned to an established working model of QoL. However, some aspects that were considered important (e. g. perceived safety) were not covered by the pre-existing domains. For that reason, we re-conceptualized the working model of QoL and added a sixth domain, referred to as *healthcare-related domain*.

**Conclusion:**

Interviewing patients and healthcare professionals brought forth specific aspects of QoL evolving in telemedical contexts. These results reinforce the assumption that existing QoL measurements lack sensitivity to assess the intended outcomes of telemedical applications. We will address this deficiency by a telemedicine-related re-conceptualization of the assessment of QoL and the development of a suitable add-on instrument based on the resulting category system of this study.

**Supplementary Information:**

The online version contains supplementary material available at 10.1186/s12955-021-01807-8.

## Background

Telemedical applications (TM) are widely used for the treatment of physical and mental illnesses. They represent a way to ensure healthcare is available to people in rural areas or during times of crisis either as a supplement or substitute to standard care.

The use of supplementary telemedical applications aims to improve patient-centered healthcare management [1–3] and targets challenges that arise in continuity of care [4]. In general, telemedical applications are defined heterogeneously [5]. In line with the World Health Organization (WHO), we understand telemedicine as “the delivery of healthcare services, where distance is a critical factor, by all healthcare professionals using information and communication technologies for the exchange of valid information for diagnosis, treatment and prevention of disease and injuries, research and evaluation, and for the continuing education of healthcare providers, all in the interests of advancing the health of individuals and their communities” [6]. In Germany, this type of healthcare is provided either by healthcare professionals in medical institutions or by commercial companies. So far, only some of the telemedical services are financed by statutory health insurers. The legal framework for the evaluation and financing of telemedical applications has only been created in recent years. Currently, digital healthcare is systematically expanding with numerous new regulations. For example, criteria for reimbursing telemedical applications are being debated and there is ongoing development of digital health technologies, including the design of more user-friendly telematics infrastructure, the promotion of digital networking, and the use of health apps in nursing care.

Patient-reported outcomes came to the fore within efficacy studies of telemedical applications, next to clinical or economical evaluation criteria [7–9]. Quality of life (QoL) became established as the most commonly applied patient-reported outcome [10]. It is assessed not only within efficacy studies, but also in the context of economic evaluations [11]. To take the impairment of physical or mental states into account [12], not only generic, but also health-related or disease-specific QoL can be measured in the context of (chronic) health conditions. Different aspects of QoL are assessable, depending on the content focus of the underlying model and the resulting instrument [13]. Reviews about the impact of telemedical care on QoL show inconsistent results [14] for most commonly addressed specific diseases like e.g. heart failure [15–21] or depression [22, 23]. Studies have applied established generic, health-related or disease-specific QOL instruments (e.g. EQ-5D, SF-36/SF12, WHOQOL-100/–BREF; [24]), that may not be sensitive enough to assess setting-related aspects of QoL in telemedical contexts. A contributing factor is that QoL assessments were designed before the use of digital treatment solutions that changed the healthcare context. Research has shown, that the implementation of telemedicine has an enormous impact on patients’ daily lives and lived experiences. A qualitative study provides evidence that telehealth is perceived as helpful in managing everyday life and enables patients to better self-manage their condition [25]. They also report that increased contact with healthcare professionals and the level of continuity of treatment enables trusting relationships to be formed over distance which alleviates feelings of isolation. Moreover, a sense of security, feelings of relief and support in self-care through access to telehealth data has been described [25]. In addition, telehealth applications can support independent living at home and controlling the health state [26]. A major gap is that patient-reported instruments sensitive for these issues are missing. So far, several patient-reported instruments applicable in the telemedical setting have been developed, e.g. for measuring satisfaction [27, 28], subjective usability [29], or patients' impressions of the risks and benefits [30]. However, none of these instruments address the assessment of QoL from the patient’s perspective in the context of telemedicine in particular.

For this reason, our study aimed to explore the impact of telemedical care on QoL of patients with chronic diseases or mental illnesses. We applied a qualitative approach to derive a concept of quality of life (QoL) in telemedical care. This concept elicitation will inform the development of a setting-sensitive instrument to assess patients’ QoL in telemedical healthcare. Until now, this is the first study to address the observed inconsistencies by challenging the adequacy of existing QoL assessments for telemedical healthcare services.

## Methods

### Study design and population

#### Sample and research context

We conducted a qualitative, observational, cross-sectional study. The participants were enrolled according to inclusion criteria, but not randomized. This study focused on expectations and experiences of patients and professionals regarding telemedical healthcare as compared to standard care and was not blinded.

The sample aimed to represent the heterogeneity of telemedical applications and patient populations to ensure more generalizable results. Therefore, we included the main groups of telemedical healthcare professionals and chose patient groups that are heterogeneous with regard to their primary disease (mental and chronic physical disease), but often included in telemedical studies. In addition, we included active (regular phone calls) and passive (remote health monitoring) telemedical applications. Therewith, we wanted to capture a variety of telemedical experiences from patients with a diverse disease, gender, age and care spectrum as well as from different telemedical professionals. The number of focus groups and interviews was chosen in order to reach content saturation [31–33] and is described in Table 1. We aimed to undertake (a) focus groups with a total number of 32 participants (patients), (b) focus groups with a total number of 30 participants (telemedicine professionals), (c) 32 single interviews with patients, and (d) 30 single interviews with telemedicine professionals. We aimed for a minimum case number of n = 30 in all groups. However, in the focus groups and interviews with patients, we included at least n = 32 participants to ensure an equal distribution of condition (physical vs. mental) and type of care (telemedical care vs. care as usual) aiming at n = 8 for each combination.

**Table 1:** Recruited sample for focus groups and individual interviews consisting of patients and professionals

Patients	Focus groups		Interviews		Total
	TM	No TM	TM	No TM	
Mental disorder	10	10	9	8	37
Chronic physical disease	9	9	8	8	34
Total (Patients)	38		33		71
Professionals	31		30		131

TM, with telemedicine; no TM, without telemedicine

All patients were recruited by the associated telemedical nurse during treatment in two university hospitals in Mecklenburg-Western Pomerania and Brandenburg, Germany; professionals were recruited nationwide via e-mail, phone or in-person contact by the first author. All eligible participants had to be 18 years or older and German speaking. Moderate to severe impairment of cognitive functions (e.g., comorbid neurological diseases) were defined as exclusion criteria. Further criteria were defined per group in terms of the disease (chronic physical or mental disease), and the telemedical experience (with or without telemedical experience). Participation in the study was voluntary; there was no disadvantage in not participating. Participants received an expense allowance. All participants provided written informed consent.

#### Data collection

As recommended for concept elicitation [34, 35], we conducted open-ended, semi-structured in-person focus groups and individual interviews with either patients or telemedical professionals. Every conversation was voice-recorded. All focus groups were led by the first author and a student transcript writer, and took place at the patients’ respective treatment clinic or in the natural work environment of the professional groups. The duration of the groups varied between 60 and 100 min. In addition, we conducted open-ended, semi-structured expert interviews. All interviews with professionals were led by the first author and were conducted in-person at a place chosen by the professional or via phone. Interviews with patients were conducted by associated telemedical study nurses at the patients’ respective treatment clinic or via phone. The duration of the interviews varied between 30 and 90 min. All participants were only interviewed once. All interviews and focus groups were conducted between July 2018 and February 2019. Finally, an expert workshop for external validation of the results was conducted as a group discussion, with six experts from the fields of TM applications and QoL research.

#### Interview and focus group guides

The interview and focus group guides consisted of mostly open-ended formulated questions and were divided into three main parts: (a) individual understanding of QoL, (b) personal description of current healthcare situation, and (c) subjective impact of healthcare on QoL. All participants could indicate not to answer a question. The different versions of the interview and focus group guides are attached in the supplementary appendix (Additional file 1: Supplementary A and Additional file 2: Supplementary B). The questions were partly adapted to the person being interviewed. Spontaneous questions for improved understanding were possible.

#### Data analysis

The recording of interviews and focus groups were transcribed word-for-word in standard German by student research assistants using the software f4transkript by audiotranskription [36]. Mayring's content analysis approach [37] was used to encode the qualitative data material with MAXQDA software [38]. The analysis aimed to identify all text sequences or units of meaning that refer to the personal meaning of QoL, the personal experience in connection with the telemedical application or standard healthcare, and its impact on QoL. At first, deductive categories were defined, that were used to structure the organization of inductive categories. The inductive categories were iteratively derived from the material by two staff members independently. After the initial coding, the inductive categories were discussed and uniformly labelled. In the following step, the material was newly assigned to existing categories independently, before the two staff members discussed the final assignment. Possible divergent codings and contradicting interpretations were discussed with a third supervising person in a consensual procedure.

#### Quality of life: a working model

QoL instruments assess different core areas of the construct: some are rather generic, while others are health-related or disease-specific. For this reason, we initially created a general working model of QoL on which we could map the results of this qualitative study. As part of a systematic literature review, we summarized telemedical efficacy studies that addressed either chronic physical or mental conditions and included QoL as primary or secondary outcome. On this basis, we identified the most commonly used generic (EQ-5D, WHOQOL-100/ WHOQOL-BREF), health-related (SF-36/SF-12/ SF-8/SF-6), or disease-specific (EORTC QOL-C30, MLHFQ, FACT) QoL instruments in telemedical efficacy studies [24]. In the next step, domains and subdomains of these instruments were analyzed. Finally, we integrated the findings on a general working model of QoL with the following domains: Biological domain, psychological domain, social domain, functional domain, and a disease-specific domain. The next paragraph describes the mapping procedure of the results of our qualitative study on this working model of QoL.

## Results

In total, 38 randomly assigned patients participated in eight focus groups of four to five participants. Patients were between 18 and 84 years old, from Northeast Germany (Federal States of Mecklenburg-Western Pomerania and Brandenburg), and of various social backgrounds. 21 patients were male and 17 female. 18 patients suffered from cardiological diseases (n=10 dilated cardiomyopathy, n=9 ischemic cardiomyopathy; n=9 each with or without telemedicine) and 20 patients had depression (n = 10 each with or without telemedical treatment). All patients received a compensation of €40 to cover expenses.

Furthermore, we conducted seven semi-structured focus groups nationwide with pre-existing working teams from a telemedical background. The teams were interviewed in their natural work environments: (a) a telemedicine unit for depression (n = 8 from university or commercial setting), (b) a telemedicine unit for heart failure (n = 14 from university or commercial setting), (c) a telemedical team in a private cardiology practice (n = 6), and (d) a start-up for telepsychiatric care (n = 3). The group size varied between three to six participants per group with a total number of 31 participants. All professionals received a compensation of €75.

Additionally, we conducted 63 semi-structured single interviews. Our participants were patients (n = 33) with chronic physical diseases (n = 16; thereof n = 9 dilated cardiomyopathy, n = 5 chronic kidney failure, n = 5 diabetes mellitus, n = 3 hypertension, n = 2 peripheral artery disease, and n = 1 rheumatoid arthritis - as most patients suffered from more than one disease. N = 8 patients each were with or without telemedical treatment). Additionally, 17 patients suffered from mental disorders (thereof n = 15 depression, n = 3 PTSD, n = 3 anxiety disorder, n = 2 schizophrenia, n = 1 panic disorder, n = 1 bipolar disorder, n = 1 substance use disorder, n = 1 personality disorder, n = 1 problem gamling, n = 1 somatic symptom disorder - as most patients suffered from more than one disease. N = 9 patients were with and n=8 patients were without telemedical treatment). All patients received a compensation of €40 to cover expenses.

Finally, we conducted semi-structured expert interviews with 30 telemedicine professionals from Germany and Austria, of which nine participants were male. The professionals came from five different areas: (a) research (n = 13), (b) provider of commercial telemedical care (n = 9), (c) telemedical care in hospitals or private practices (n = 6), (d) politics (n = 1), and e) health insurance companies (n = 1). All professionals received a compensation of €75.

### Treatment of patients in the telemedical group

Patients with mental disorders received telephone support in addition to standard treatment. A telemedical contact person called the patient at individually defined times in variable intervals for an average of 30–50 min. At the beginning of each telephone call, standardized questionnaires were used to document the course of the disease, followed by a discussion of individual topics. Patients were able to reach their telemedical contact person in an emergency. Patients with chronic physical illnesses were integrated into a telemedicine system, and received an electronic scale to take home as well as a digital device that automatically sends data to their hospital. After an introduction, patients were asked to weigh themselves every morning at home. If the automatically transmitted values exceeded a predefined tolerance range, the patients were contacted by a heart failure nurse and, if necessary, further steps were taken to manage the situation (e.g., making doctor's appointments, adjusting medication). Patients had the possibility reach their telemedical contact person in case of an emergency.

### Derived conceptual framework

In the following section, we describe various facets of QoL domains that study participants referred to and give examples of how they are impacted by telemedical healthcare. A quantitative summary of the data evaluation can be found in supplementary appendix (Additional file 3: Supplementary C). Participants’ quotes are highlighted with italic formatting. They were slightly edited within the translation process for improved comprehensibility.

### Pre-existing domains

#### Biological domain

According to the participants, sleep and pain are crucial aspects of QoL that can be assigned to the biological domain *(“I also have other problems where I have a very poor quality of life: For example, I can walk twenty meters without pain. Above twenty meters I have pain in my calves. Above forty meters it becomes unbearable.”)*. We conclude from the data that telemedicine impacts those two essential aspects, for example by monitoring the patients’ symptoms, by helping them increase their health literacy, and by adapting clinically rational medication based on increased availability of data.

#### Psychological domain

In the context of the psychological domain of QoL, the facets of psychological *well-being, mood, cognitions,* and *self-esteem* play a decisive role in everyday life with chronic physical or mental diseases. *Psychological well-being* comprises aspects like fear, self-care, meaning and perspective, vulnerability as well as the feeling of being left alone with the disease *(“I don't go out alone anymore because I am afraid. I get dizzy more often and that's why I'm so afraid to go out on the street alone and my husband has been dead for 26 years, I have no one else.”)*. It can be improved by telemedicine through increased health literacy and knowledge about the disease and treatment options (“It is clearly the content that has an influence. The content is also taught in outpatient therapy. But I also believe that digital medium plays a very important role. The user has to become active, which creates an additional therapeutic effect. / I think the patient is more likely to become an educated patient, that he*she understands himself*herself and his*her disease or health condition better, that he*she gets a better feeling and can act more at eye level with the doctor.”). Moreover, patients appreciate the opportunity of talking to a neutral contact person from telemedical personnel to discuss fears and issues that concern them *(“The moment we have a phone conversation and I can tell my problems, I feel better already.”).*

If we look at *mood*, it is noticeable that many respondents associate positive mood with QoL, but often suffer from negative mood and feelings (e.g. frustration) in the context of their disease and the associated treatment *(“I observe depressive moods more often. I am not depressed per se, but I immediately view everything negatively without any plausible reason. (…) This accompanies me much more strongly in my life than when I still had a healthy heart.”).* According to the reports, telemedicine is a way to improve mood and can help to deal with negative feelings: Applications can improve it by assisting with questions, difficulties with treatment, disease management, or topics from everyday life *(“It's fun talking to the telemedicine nurse. I tell her something and she can give me advice on how to handle a situation better.”).* In addition, communication between telemedicine personnel and patients can have a distracting and relaxing effect. Lastly, some patients simply enjoy using telemedicine *(“When I know that the telemedicine nurse is calling, I lie down on the couch and take the phone with me. It's really nice and relaxed. Not as stiff as with the psychologist.”).*

Negative thoughts, indifference, and guilt shape the statements that can be assigned to the facet of *cognitions (“For me, quality of life is to be able to get up in the morning without carrying negative thoughts all day.”).* This is addressed by telemedicine through additional communication, shared reflection processes, and symptom management *(“In our program, an important part is needs and goals in life. People actively deal with how they actually want to live. At that moment, they already reflect on what they spend their time on, what they want to spend their time on, what they want to change. It can be a change in private life, so that one takes more time for positive activities, for family and friends, for self-care. And at the same time also at work, e.g. problem solving is often an issue.”).*

Finally, it was described that *self-esteem* can be reduced by chronic diseases. Patients report they feel less valuable or that they are a burden for others due to their disease *(“It's such a burden, it's so stupid, I'm burdening my husband with it.”).* Here, telemedical applications can increase the self-efficacy experience of patients with regard to their disease and coping with their everyday lives *(“Quality of life of depressive patients means they can experience self-efficacy despite their illness. Be it in social contact, be it in a professional or voluntary context, or even in sports activities or creative pursuits.”)*. It is crucial that patients feel competent in dealing with their own disease. Moreover, therapy and disease management can be simplified, e.g. by providing distant treatment so that patients do not have to rely on help for transport. Simplifying care can help patients to perceive themselves as less of a burden on their relatives. Therefore, information should be tailored to patients’ current life situation. Additionally, patients should receive support in disease management and suitable adjustments of the type of treatment. Finally, the communication between patient and telemedical personnel seems to build self-esteem *("Did you have any expectations about the telemedicine care beforehand?"—"No expectations, because I didn't yet know what was in store for me. (…) From today's point of view, I have to say that it is very positive, I experience it as constructive for me, stimulating. And above all, my self-esteem is strengthened again, particularly when things are going badly for me.").*

#### Social domain

With regard to the social domain of QoL, study participants stated that *social relations, support, norms,* and the *environment* play an important role. They describe that the disease’s impact can lead to avoidance behavior that impedes socializing or maintaining contacts, and often leads to social isolation, which harms the patients’ QoL. In contrast, the existence of relatives or friends is experienced as beneficial. Telemedical treatment can address the effect of feeling socially isolated as it often provides an additional contact to communicate with *(“Well, even if you're alone, like I was, and I was always single in between, you're not left alone. You don't sit alone and kill yourself because there's no one there to stop you, right? (.) They call me every week. You didn't even get to kill yourself.”)*. Unlike with family and friends, the relationship to the telemedical personnel is mostly unidirectional with the patient’s needs in the center of attention, and no expectation of reciprocity. While regular telemedical contacts can disburden private contacts when patients can communicate about their disease with competent staff, private contacts of the patient can also be involved in the treatment, for instance in educational sessions or conversations about everyday life challenges.

Study participants describe perceived *social support* as beneficial. However, it is often missing due to social isolation or social contacts being helpless *(“When I’m open with the people around me and say that I am not doing so well, and tell them what is not going well, my problems, I felt it puts people in a position that very few people can handle and want to handle.”).* Consequently, patients perceived it as supportive to stay in touch with competent telemedical staff that can provide help for coping with everyday life. As such, a regular contact to the telemedical staff can partly compensate for missing social support by patients’ private contacts.

Third, *social norms* play a role for the interviewees in evaluating their QoL. A perceived pressure to perform was described, which often arose from the comparison with other (healthy) individuals. In addition, they noticed a lack of societal sympathy for the disease’s symptoms or the treatment’s side effects, and often felt misunderstood. Finally, some of the patients reported to be responsible for partners or a children in need of care, and that they find it difficult to deal with the feeling that they cannot always live up to this responsibility because of their disease. To reflect the self-image, the perceived pressure, and to find solutions for challenging situations via telemedicine can often relieve patients. Again, a regular and competent contact can support coping processes, educate about disease management skills or tools, and make everyday life more livable.

Finally, the *social environment* has an impact on the participants’ QoL. As such, patients described it as positive to be in pleasant surroundings and live together with people they love and appreciate. As telemedicine can be brought to the patient, it supports the desire to be treated in a familiar environment.

#### Functional domain

In the context of the functional domain of QoL, the facets of *autonomy, general level of function*, and *level of activity or participation* play a decisive role in everyday life with chronic diseases.

*Autonomy* was described as the ability to meet basic needs, to handle the everyday tasks independently, to be mobile, and to manage one’s own daily schedule *(“I am afraid of becoming more and more of a burden for others. That's in the back of my mind, it is terrible. I have always been active, I have had four children and raised a grandchild. (…) With many, many things I am now dependent. It's so terrible, unbelievable.”).* It is also understood as having financial resources or property, and the option to travel and go on vacation *(“I am very proud of the fact that I am now working again and can therefore afford a car again.”)*. However, patients suffering from a chronic condition often face limited possibilities in managing their everyday life independently, and the extent of their autonomy is often linked to the severity of the disease. Telemedicine can be used to improve patients’ autonomy in several aspects: Firstly, it can provide location-independent healthcare which is also accessible for immobile patients, and it saves travel costs and efforts *(“What patients mentioned repeatedly: Many of them did not dare to leave their homes anymore. Travelling were not possible because they somehow thought, 'Well, if something happens, I have to get to my cardiologist or to the hospital quickly'. Now that they are supported by telemedicine, they can take their device with them and “have the doctor in their pocket”. That way, patients can go on a trip again.”).* Secondly, some telemedical applications can be used flexibly with regard to time and duration while others provide daily orientation and therewith a certain stability in everyday life *(“What I really appreciated about telemedicine (…) was that the length of the telephone call was always based on my needs. I determined the length. When I was feeling bad, the call was longer, and when I was feeling better, the call was shorter. I found that very, very nice compared to outpatient therapy.”)*. In conclusion, telemedical treatment may be better integrable. Thirdly, telemedicine may provide help for self-help and guidance within the everyday context to increase autonomy in a real-life situation.

The *general level of functioning* influences QoL *(“Sometimes I feel like my mind is still young, but my body no longer works well and that makes me sad, it hinders me. You want more than you can actually do.”)*. For instance, being able to work, maintaining a structured daily routine, work-life-disease balance, and the degree of avoidance behavior were described as crucial. Telemedical applications may help in symptom management and provide help for self-help. Continuous treatment supports patients in structuring and organizing their day. Finally, guided stimulation of exposure, followed by a reflective process may help to improve the general level of functioning.

The *level of activity or participation* comprises physical and mental participation, career opportunities, hobbies, and sports *(“Sure, it is important for the quality of life to pursue one's own needs and hobbies as well”).* A higher level of participation was described as beneficial for the perceived QoL. However, many patients feel limited by their disease. Telemedical applications may improve the level of activity by providing support in symptom management, help for self-help, and guided participation (“Activation is simple, the patient gets up, turns on the tablet, answers his*her questionnaire, maybe even listens to his*her inner self, which can be positive. Of course, he*she is also activated by various things: We included sports programs and pedometers that motivated the patients, we provided recipes where the patients say: ‘Man, I haven't tried that yet’, and they go out and buy ingredients that they have never worked with before. He*she expands his*her knowledge, his*her spectrum and attention.”).

#### Disease-specific domain

According to study participants, the *impact of the disease, disease-related environmental factors*, and the *acceptance of the disease* are key elements that influence QoL.

The perceived *impact of the disease* was described by the interviewees stating limitations due to symptoms *(“Quality of life for me is to live as I lived before the disease. Of course, I also have to admit to myself that I can no longer do everything the way I did before. But I still want to do as much as I can.”)*, physical as well as mental effects of the disease, the stability of the course of the disease and sometimes even a limited life expectancy. Most importantly, telemedical treatments should support the monitoring, limitation, and management of symptoms, and accompanying the patient as emotional support.

Moreover, *disease-related environmental factors* play an important role: Handicapped accessible means of transport, inner-city infrastructure (e.g. public toilets), or easy-accessible medical facilities are appreciated, whereas the lack of these leads to tremendous effort on the side of the patient, or avoidance behavior. Even though telemedicine cannot change the social environment of the patient, it can make the treatment more and easier accessible, as it can be brought to the patient’s home or place of choice via information and communication technologies.

Finally, the *acceptance of the disease* and the (self-) destigmatization are important processes that can change QoL in a patient *(“And you simply have to realize that you have to allow yourself these breaks. If you're sick, you're sick, that's just the way it is.”)*. At that point, it is appreciated if telemedicine supports through communication, education, and the exchange of experiences. In addition, telemedicine can broaden the access to care *(“We also know that there are groups of patients who would not dare to go to a psychiatric clinic for fear of stigmatization. Telemedicine services can also help these patients to access care”).*

### Model extension: new findings based on our qualitative studies

The majority of aspects that influence the QoL of patients dealing with chronic conditions or mental illnesses could be assigned to the identified working model. However, some aspects that were considered important were not covered by the pre-existing domains yet. For that reason, we extend the working model of QoL and added a sixth domain to it, referred to as *healthcare-related domain*.

#### Healthcare-related domain

**Fig. 1:**
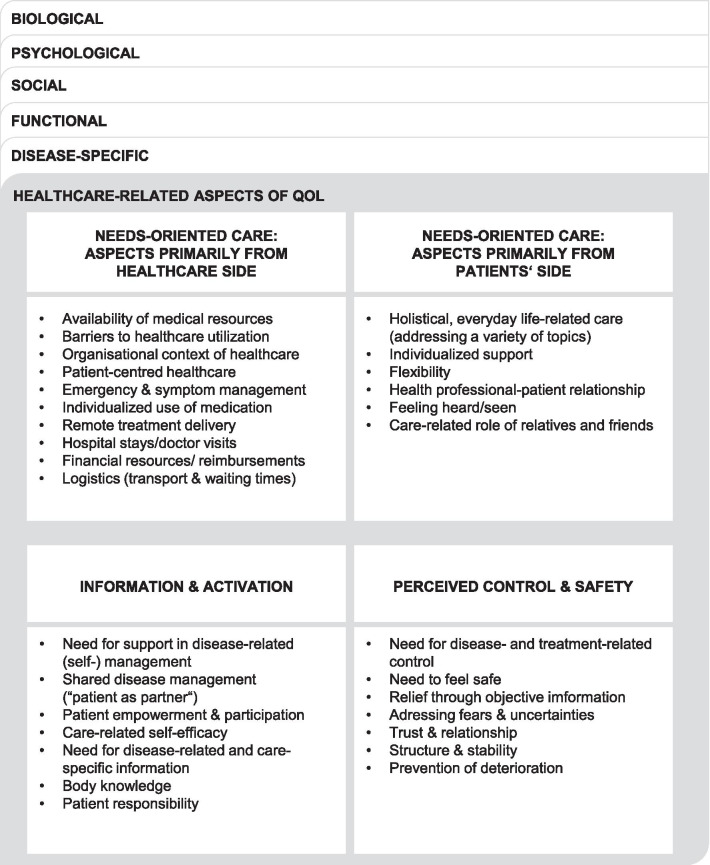
Healthcare-related domain

The healthcare-related domain summarizes healthcare-related aspects that increase or decrease patients’ QoL. It comprises four facets: (a) needs-oriented care: aspects primarily from healthcare side, (b) needs-oriented care: aspects primarily from patients’ side, (c) information and activation, and (d) perceived control and safety (Fig. 1).

### Needs-orientated care: aspects primarily from healthcare side

Firstly, organizational structures influence *needs-oriented care*. Limited available treatment resources and bureaucratic barriers were reported (e.g. required letters of referral). Patients often face a high number of doctor visits or hospital stays, which involves many journeys, long waiting times, and financial resources. Compared to standard care, they desire more patient-centered care that supports symptom limitation and quick emergency management *(“When I don’t feel well, it’s very difficult to start a conversation to get help right away. It's an enormous relief for me that I can call the telemedicine first. Sometimes, the doctor doesn't have time right away and then it's good that you first have a contact person with whom you can talk until you have an appointment with the specialist.”)*. Study participants report that using telemedicine can help to improve needs-oriented care aspects that are primarily given by existing healthcare structures: As such, telemedicine is experienced as an easier way to access continuous treatment by an often multi-professional team that is connected within a network of care. Patients undertake fewer journeys due to the location-independent treatment and experience fewer waiting times. Telemedicine enables quicker therapy adjustments and support of patients to achieve therapy goals in everyday life, including emergency aid. Complementary telemedicine can compensate for limited medical/therapeutical in-person resources and provide an efficient healthcare solution for both professionals and patients. Some patients report that digital treatment is helpful for the treatment process, as it seems to be easier to be honest about sorrows and non-compliance in a non-face-to-face setting. Finally, healthcare professionals who actively use telemedicine report better justified adaptation of medication based on long-term monitoring, and sometimes even a reduction of drugs.

### Needs-orientated care: aspects primarily from patients’ side

Secondly, there are *needs-oriented aspects* that primarily arise from the patients and their living environment. These comprise different types of “relationships”, like the relationship between healthcare personnel and patient. It is described as beneficial for individualized support if the telemedical personnel have a certain understanding for the everyday life of the patient. This closeness often leads to the patients feeling heard and seen. The other QoL-relevant relationship is the role of relatives, friends, partners or significant others in care-giving. The additional role as a caregiver often leads to a plethora of feelings for the patient (e.g. appreciation, guilt, or happiness) and the caring person (e.g. helplessness, excessive demand, or hope). A chronic condition or mental illness alters the relationship, which is experienced as challenging. More than regular care, telemedicine usually provides flexible possibilities to communicate with other concerned individuals or competent staff via phone, e-mail, or (video) chat. Patients and professionals stated to experience these instances of communication to be more at eye level as compared to traditional patient-doctor conversations *(“As nurses, we naturally talk to patients differently than, for example, specialists. So you go into the conversation with a different vocabulary.”*). Patients appreciate the tone and continuity of communication. Patients and healthcare professionals highlight the freedom for individuality within some telemedical applications, and that patients benefit more from consistent care than from one-off doctor’s appointments. Additionally, patients appreciate the opportunity to not only communicate about their condition and the treatment itself, but also about everyday life challenges that come along with it *(“What I like about telemedical care is that you can talk about all problems. One's own needs are specifically addressed.”)*. As a result, the telemedical communication is perceived as relieving. Some telemedical applications, such as regular phone calls by medical staff, are characterized by the consistency of a contact person, so that a bond and trust between the patient and the contact person can be built over time despite spatial distance. This often leads to increased honesty and willingness to discuss challenging topics, which can also benefit other, private relationships. It is not uncommon for relatives to be involved in telemedical care, too, for example to clarify questions. Finally, the flexibility with regard to time and location makes the practical treatment easier for both the patients and their social environment.

### Information and activation

Thirdly, *information and activation* influence QoL. Patients and professionals describe it as the patients’ need for knowledge about their specific disease and treatment, and sustained support for managing their condition. Education further enables patients to take on responsibility for their health-related behavior and to self- or co-determine treatment decisions *(“Patients have the daily task of recording their vital signs. This already triggers something in many people because they have a feeling that they have made a contract with us and they feel responsible for fulfilling it. (…) Patients become more aware of what a certain behavior does to their body, and this also strengthens their personal responsibility again.”)*. Professionals described the process to be most effective when healthcare professionals strongly guide disease management first, and then empower the patient stepwise to become an expert for their own body, mind, and condition—as far as possible. This process also promotes the development of care-related self-efficacy in patients or their social environment *(“We receive feedback from the patients, or via therapists about their patients, in the form of quotes such as 'I managed that, I worked hard for that'. Therapists who work with patients in only face-to-face scenarios tend to get feedback like, 'I could never have done that without you.' So the success of the therapy is attributed a lot to the therapists, and in online therapy it is more often the case that the patients actually experience that they have certainly worked hard themselves to reach their goals.”).* Both patients and healthcare professionals described that telemedicine is a way to empower patients’ own disease management and thereby strongly improve QoL. As in traditional care situations, telemedical patients get information about their disease and about different treatment options. However, telemedical applications provide an active or passive guidance for patients in their daily lives, which goes far beyond one-off doctor’s appointments. Consequently, patients can train newly learned health behavior or disease management skills, ask questions, and clarify misunderstandings in a simplified manner. Furthermore, patients appreciate the continuity of guidance and help for self-help, the consistency of a contact person, and the possibility to co-determine treatment decisions within the telemedical context. Lastly, patients and professionals appreciate the constant awareness about the course of the disease through objective data monitoring as an additional source of information *(“We have observed that patients gain more peace of mind in the daily management of their disease by knowing that a health professional has the possibility to view patient-related follow-up data. This knowledge alone has a major effect. (…) Patients feel safer, which is an essential component of improving the quality of life.”)*.

#### Perceived control and safety

The fourth facet that influences QoL was named *perceived control and safety*. It is defined by statements from patients and professionals about how a disease can make the patient feel vulnerable in their daily life due to fears, lack of knowledge, uncertainty, or treatment intransparency. Primarily, patients describe to feel “relieved through certainty”, which means they feel better after a doctors’ appointment, because the doctor makes statements about the disease and the patient’s state of health. An expert’s opinion can satisfy the need for control and safety, but is often missing in between scheduled medical check-ups. Patients and professionals stated that the needs for control and safety can be better addressed in the context of telemedical treatment than in a care-as-usual context: The frequent monitoring of (objective) health-related parameters gives patients the feeling of structure and control *(“It is reassuring to know that the device would react and call the hospital in case of an emergency.”).* Often, telemedicine enables patients to monitor their disease and check their symptoms by themselves whenever they want. In addition, low-threshold follow-up care and prevention, e.g. by monitoring symptoms, can prevent worsening of the disease. Additionally, patients can often also get quick and direct professional feedback through active or passive guidance by telemedical personnel. Contact with socially and medically competent telemedical staff can build trust through a relationship experience, which can further reduce fear and uncertainties and increase the feeling of being supported. Hence, the decisive advantage of telemedical care lies in continuous care in the daily lives of patients and the possibility to quickly communicate with telemedical staff. Further, telemedicine is described as beneficial to bridge the time between a hospital stay and the next doctor’s appointment being back at home *(“It was like a little stepping stone: You still felt safe and you still had such a slight connection to the clinic. I found that very helpful.”).*

Some disadvantages of telemedical care were reported by a few patients. Some participants question the data processing and privacy protection of telemedical systems, while others even feel “spied on” by telemedical systems *(“Some patients were afraid of surveillance or felt they were under surveillance because of the questionnaires. They did not take part in the study or became drop-outs”).* These doubts for example can be resolved with the help of data-related information (e.g. data protection statement), technical introductions, and a high degree of transparency in order to increase utilization of and satisfaction with treatment.

## Discussion

The assessment of patient-reported outcomes such as QoL plays a decisive role in evaluating and optimizing telemedical applications—and thus everyday care in the future for millions of patients. This qualitative study examined the impact of telemedical applications on QoL from the perspective of chronic physically or mentally ill patients, as well as telemedical healthcare professionals. As a result, we mapped the resulting category system on a working model of QoL, consisting of five widely established domains. Our results suggest that telemedical applications influence the patients’ QoL and that this impact is not fully covered by existing domains, yet. Therefore, we summarized the unmapped aspects stated by the participants and conceptualized them as a sixth QoL domain, referred to as healthcare-related domain. From a conceptual perspective, this domain is associated with already established domains integrated in existing operational models of QoL and related to the provision of healthcare, such as impact of “treatment” or “medication”. However, telemedical applications transcend such treatment-specific QoL approaches, as they shape a principal new kind of healthcare delivery and have some essential characteristics in common (e.g. use of ICT technology, absence of medical professionals).

### Relevance

The increase in chronic physical and mental illnesses is changing the role of treatment. As a result of medical progress, we are able to live with a disease and therapy for longer periods of time. The treatment of a disease therefore plays a crucial, even everyday role in the lives of those affected. It is no longer a matter of merely regulating symptoms. Rather, the influence of treatment on the individual and his or her environment must be considered holistically. Aspects such as organizational structures of care, the patient's development of competences, the relationship with healthcare professionals, and the inclusion of the social environment, time expenditures, and emotional as well as financial burdens are increasingly receiving attention. Now it seems necessary to extend the existing QoL concept in order to take into account the special features of the treatment context in the evaluation of telemedical applications compared to standard care. A specification of the assessment context has been successfully achieved in the past with regard to the development of disease-specific instruments. We now propose a broadening of the perspective, in which not only specific aspects of a disease, but also its treatment setting is considered as variable influencing QoL.

### Integrating study results and previous research

Our findings are consistent with previous research, indicating that most of the facets and categories mapped onto the healthcare-related domain were also found to be important in other qualitative studies within the context of telehealth:

#### Needs-oriented care

In a study about patient experiences to osteoporosis care delivered virtually by telemedicine, Palcu et al. describe “convenience of timely care close to home as well as a reduction of burden of travel and costs” [39] as benefits of telemedicine, which is in line with our results. Powell et al. [40] state benefits with regard to convenience and costs, too, adding that the patients can be in their own supportive environment during the treatment as another advantage. Brunton and colleagues [25] conducted a qualitative meta-synthesis about telehealth user experience in COPD. They found out that telehealth was perceived as helpful in managing everyday life and enabled patients to self-manage their condition. They also report that increased contact to healthcare professionals and the level of continuity enables trusting relationships to be formed which alleviated feelings of isolation. In addition, many telehealth solutions are designed in a way that family members become more actively involved. This qualitative meta-synthesis further supports our findings. However, only Lee et al. [26] related constructs of needs-oriented care to QoL: As such, easy access to the doctor and convenient healthcare services are perceived as important components for improving quality of life.

#### Information and activation

In a study about hip fracture patients’ experiences with testing an app, Jensen et al. [41] reported that telemedical applications are a way to support information and education for patients and hence address individual learning and health literacy needs. They proved in an elderly sample that an app has the potential to support the ability to perform self-care and the desire for autonomy. Therefore, empowering patients seems to be crucial. According to Clemensen et al. [42] patients will have a more dominant role in taking care of their own health against the background of demographic change. Brunton et al. [25] describe similarly that patients play a more active role in their care e.g. by taking on monitoring of symptoms. By becoming more involved in managing and shared decision making, patients develop a stronger sense of accomplishment with regard to their health outcome. Lee and colleagues [26] explained that patients using telehealth for type 2 diabetes management perceived telehealth as help to live independently at home and to “be in more control over their own health state” [26]. All these described components could be retrieved from our qualitative study, too, and are integrated within the facet information and activation.

#### Perceived control and safety

Aspects relating to the facet perceived control and safety were discussed in a qualitative meta-synthesis by Brunton and colleagues [25]. Telehealth “provided patients with a sense of reassurance and a strong sense of feeling ‘looked after’” [25] through increased contact between patient and healthcare-provider as well as the knowledge that the health data is being remotely monitored. They describe a “sense of security” [25] reported by study participants due to regular contacts and through access to telehealth data. Moreover, a sense of relief and the feeling of being supported in self-care was stated. Also negative, intrusive aspects of telemedicine were reported: Powell and colleagues [40] describe that some participants in a study about patient perceptions of telehealth primary care video visits had concerns about privacy of the conversations. In our current study, this aspect is captured within the facet of perceived control and safety and can be linked to the privacy dimension of the obtrusiveness concept by Hensel et al. [43].

### What this study adds to the literature

By mapping the qualitative results to a general working model of QoL, it was shown that there are relevant patient-reported constructs that are not yet represented by the concepts of the existing instruments (summarized within the healthcare-related domain). For the most part, these constructs also play a role in standard care and some have already been examined in other telehealth studies, e.g. empowerment [41] or perceived safety/sense of security/reassurance [25]. Nevertheless, there is no integrated concept of these constructs with regard to their effect on QoL of patients. Thus, the extension of previous QoL concepts described in this study represents an attempt at conceptual integration to fill this research gap. Finally, our study implies that existing QoL instruments are not comprehensive enough for the context of telemedical care, whereas existing telemedicine-specific instruments are not dedicated to measuring QoL.

### Is this QoL we are talking about?

Some of the aspects described by patients and healthcare professionals, which we summarized as a complementary healthcare-related domain, are already known from previous discussions and other healthcare contexts. Examples include patient satisfaction, patient empowerment, or perceived safety. Consequently, would it not make sense to simply use existing instruments of these constructs in evaluations of digital applications? This would certainly be a good first step forward making the evaluation of digital applications more patient-centered. However, we are more concerned with the question of whether it is legitimate to combine the identified constructs into a sixth QoL domain. One could argue that we simple describe the interaction of the environment with disease-specific aspects like symptoms, and the patient’s functional status [44, 45]. Certainly, the healthcare-related domain interacts with established domains of health-related and disease-specific QoL. However, these do not adequately cover aspects reported by study participants. Our qualitative study provides evidence that the aspects of the healthcare-related domain have a clear impact on patients’ QoL, as they were independently stated when asked about the individual understanding of QoL and whether or not treatment affects it. In terms of patient orientation, we should bring more attention to the fact that patients refer to these aspects as belonging to their QoL than to rely on pre-existing conceptual thought patterns. As a consequence, we should generally reflect on our traditional concepts against the background of a patients’ state of conditional health and innovative treatment application—our proposal for the extension of the QoL working model in context of telemedical care is a first step in this direction.

### Strengths and limitations

The strength of this study is the qualitative deductive-inductive approach including complementary groups (chronic physical and mental illness; active and passive telemedical approaches; patients and healthcare professionals). The resulting data does not only inform the research question, but also provides the basis for item generation of the “add-on” patient-reported outcome instrument we are aiming to design. Thus, we meet the call for contemporary PRO instrument development [34, 46]. Finally, our data is characterized by high content validity and a large sample size. The limitations of the study relate to the implementation, the selection of included telemedical applications, and language issues. First, we cannot determine what difference it made to study participants whether the interview is conducted by a study nurse or a research assistant. In addition, we included only those telemedical applications in our study that are used to complement, not replace, standard care. Third, the landscape of telemedicine is very heterogeneous. For this reason, the results presented here are not generalizable to all other telemedical applications. Finally, all data were collected in the German language and therewith also may reflect some content specific to a German context.

### Conclusion and outlook

Two main points can be derived from the results of this study: First, the complementary use of telemedical applications can lead to an improvement in patients' QoL—but only if it is meaningfully integrated into everyday care and developed together with patients and healthcare professionals in order to meet their healthcare needs. Second, to evaluate whether telemedical applications have an impact on patients' QoL, suitable instruments must be used. Existing QoL instruments are not sufficiently context-sensitive for this purpose. Because the impact of the healthcare-related domain is not covered by existing instruments yet, we will develop an “add-on” questionnaire to use in addition to traditional QoL instruments in the context of evaluating telemedical applications. The qualitative data from this study is used for concept elicitation and serves as a pool for item generation. This newly developed instrument shall help to generate reliable evidence within the evaluation of telemedical applications. Herewith it will not only support e.g. health insurance companies to evaluate and fund telemedical applications, but also patients and professionals to benefit from innovative additional care.

## Supplementary Information

**Additional file 1.** Supplementary A: Focus group guides.

**Additional file 2.** Supplementary B: Interview guides.

**Additional file 3.** Supplementary C: A quantitative summary of the data evaluation.

## Data Availability

Not applicable.
