# Supplementary material for: Same same-but different: using qualitative studies to inform concept elicitation for quality of life assessment in telemedical care: a request for an extended working model
Source: Health Qual Life Outcomes. 2021 Jul 5;19:175. doi: 10.1186/s12955-021-01807-8 (PMC8256487; doi:10.1186/s12955-021-01807-8)
Supplement: Supplementary file 1 — Additional file 1. Supplementary A: Focus group guides. [file 12955_2021_1807_MOESM1_ESM.docx]

*Focus group guide: Patients (with or without telemedical treatment experience)*

1. **Presentation of the participants & the aim of the focus group process (anonymous)**
2. **Subjective understanding of Quality of Life**

- What do you understand by the term *Quality of Life*?
- Please think freely: What does Quality of Life mean for you personally?
- Not mandatory: Can you agree on a description as a group?
- What role does health play in evaluating your Quality of Life?

1. **Description of current healthcare situation**

- Please describe your current (telemedical) healthcare situation!
- Imagine someone has never heard of your treatment. How would you describe it to him or her?

If not yet described independently, please ask the following questions:

- - Why was your current (telemedical) treatment subscripted?
  - Who is involved in your (telemedical) treatment?
  - Where does your (telemedical) treatment take place?
  - How many doctors do you have to visit because of your disease?
  - How often do you have doctor’s appointments?
  - Which documents do you need for a doctor’s appointment?
  - How long do you wait for a doctor’s appointment on average?
  - How long does a typical doctor’s appointment take?
  - Do you need additional aids to be able to carry out or follow the treatment (e.g. technology)?
  - What do you do in case of an emergency related to your disease?
- What are your expectations of your (telemedical) treatment?
- How has your treatment affected your everyday life?
- How do you experience your treatment?

Examples:

- - Do you have confidence in your treatment?
  - Do you doubt your treatment?
  - Do you feel safer/ more insecure through your treatment?
  - Do you perceive an extension or limitation of your possibilities by your treatment?
- How do you evaluate your current treatment?
- Would you recommend your treatment to a friend?

1. **Impact of healthcare on Quality of Life**

- In which way has your disease affected your Quality of Life?
- Which impact does your healthcare have on your Quality of Life?
- How is your Quality of Life affected by your current (telemedical) treatment?
  - Which areas of your Quality of Life are affected? Do they improve or worsen?

*Focus group guide: Professionals*

1. **Presentation of the participants & the aim of the focus group process (anonymous)**

- Professional background, telemedical experience, target patient group

1. **Understanding of Quality of Life**

- What do you understand by the term *Quality of Life* from a personal perspective?
- What do you understand by the term *Quality of Life* from your professional perspective?
- What do you think your patients understand by the term *Quality of Life?*
- In your eyes, which areas of Quality of Life are affected in your patients due to the disease?
- In your opinion, what should be done to improve Quality of Life of your patients?

1. **Description of current healthcare services provided by the focus group participants
   (natural working team)**

- Please describe your current (telemedical) healthcare situation!

If not yet described independently, please ask the following questions:

- - Which type of telemedical care do you provide?
  - Which application/ technology do you use?
  - Which professions are involved in the implementation of your telemedical care?
  - Did you have to get additional qualifications in order to provide telemedical care?
  - How has the use of telemedicine changed everyday working life?
  - How does it feel for you to treat patients from a distance?
    - Do you feel safer/ more insecure?
    - Do you feel more/ less supportive?
  - Who is your target patient group?
  - What are you aiming at with the use of telemedicine?
  - What do you expect from the use of telemedicine?
  - How does telemedical care affect patient care?
  - What experiences have you had so far with the use of telemedicine?
  - Has quality of care changed through the use of telemedicine?
  - To which patients would you (not) recommend treatment with telemedicine?
  - How do you evaluate the use of your telemedical application so far?
  - How is your telemedical care funded?

1. **Impact of telemedical healthcare on Quality of Life**

- How does the/ your telemedical healthcare affect Quality of Life of your patients?
- Which impact does/ could telemedicine have in general on the Quality of Life of patients with chronic conditions?
- In addition to the aspects you have mentioned, are there any other patient-reported outcomes that are affected by telemedicine?
