# Supplementary material for: Same same-but different: using qualitative studies to inform concept elicitation for quality of life assessment in telemedical care: a request for an extended working model
Source: Health Qual Life Outcomes. 2021 Jul 5;19:175. doi: 10.1186/s12955-021-01807-8 (PMC8256487; doi:10.1186/s12955-021-01807-8)
Supplement: Supplementary file 2 — Additional file 2. Supplementary B: Interview guides. [file 12955_2021_1807_MOESM2_ESM.docx]

*Interview guide: Patients without telemedical treatment*

1. **Introduction and education**

- Welcome and thanks for participating
- Presentation of the project and the interviewer
- Goal of the interview
- Information about the course of the interview, ethical education
- Clarifying questions

1. **Introduction of the patient**

- Sex/gender
- Age
- Partnership status and children
- Employment status
- Disease
- Experience with telemedical care

1. **Description of current healthcare situation**

- How would you describe your disease?
- Which impact does your disease have on your everyday life?
- Imagine someone has never heard of your treatment. How would you describe it to him or her?

If not yet described independently, please ask the following questions:

- - Why was your current (telemedical) treatment subscripted?
  - Who is involved in your (telemedical) treatment?
  - Where does your (telemedical) treatment take place?
  - How many doctors do you have to visit because of your disease?
  - How often do you have doctor’s appointments?
  - Which documents do you need for a doctor’s appointment?
  - How long do you wait for a doctor’s appointment on average?
  - How long does a typical doctor’s appointment take?
  - Do you need additional aids to be able to carry out or follow the treatment (e.g. technology)?
  - What do you do in case of an emergency related to your disease?
- What are your expectations of your (telemedical) treatment?
- How has your treatment affected your everyday life?
- What kind of relationship have you developed with the professional staff of your current treatment?
  - Is it important to you to build a relationship with the professional staff?
- How do you experience your treatment?

Examples:

- - Do you have confidence in your treatment?
  - Do you doubt your treatment?
  - Do you feel safer/ more insecure through your treatment?
  - Has the treatment changed your feeling of responsibility for your health?
  - Has the treatment changed your feeling of personal autonomy?
  - Has you perceived an extension or limitation of your possibilities by your treatment?
  - Has the treatment changed your awareness of your symptoms or condition?
- How do you evaluate your current treatment?
  - What are benefits of your current treatment?
  - What are disadvantages of your current treatment?
  - Are there any aspects of your current treatment that you would like to improve?
- Would you recommend your treatment to a friend?

1. **Quality of Life**

- What do you understand by the term *Quality of Life*?
- Please think freely: What does Quality of Life mean for you personally?
- Which areas of your life are particularly important for your Quality of Life?
- What role does health play in evaluating your Quality of Life?
- How has your disease affected your Quality of Life?
- What kind of impact does your current treatment have on your Quality of Life?
  - Which areas of your Quality of Life are affected?
  - Which of the stated areas improved? Which worsen?
- What would have to change in order to improve your Quality of Life?

1. **Telemedicine**

- Have you ever heard of Telemedicine?
- What do you imagine Telemedicine to mean?
- Would you try this type of treatment?
- What concerns would you have in the run-up to the telemedical treatment?
- What expectations would you have of telemedicine?
- What do you think: Would the telemedical treatment affect your everyday life?
  - What could be benefits of telemedical treatment?
  - What could be the disadvantages of telemedical treatment?
- What do you think: What would be the main difference between the telemedical treatment and your current treatment?
- What do you think: Would the telemedical treatment affect your Quality of Life?

Finally, is there anything you would like to add about your disease, treatment or Quality of Life?

*Interview guide: Patients with telemedical treatment*

1. **Introduction and education**

- Welcome and thanks for participating
- Presentation of the project and the interviewer
- Goal of the interview
- Information about the course of the interview, ethical education
- Clarifying questions

1. **Introduction of the patient**

- Sex/gender
- Age
- Partnership status and children
- Employment status
- Disease
- Experience with telemedical care

1. **Description of current healthcare situation**

- How would you describe your disease?
- Which impact does your disease have on your everyday life?
- Imagine someone has never heard of your treatment. How would you describe it to him or her?

If not yet described independently, please ask the following questions:

- - Why was your current (telemedical) treatment subscripted?
  - Who is involved in your (telemedical) treatment?
  - Where does your (telemedical) treatment take place?
  - How many doctors do you have to visit because of your disease?
  - How often do you have doctor’s appointments?
  - Which documents do you need for a doctor’s appointment?
  - How long do you wait for a doctor’s appointment on average?
  - How long does a typical doctor’s appointment take?
  - Do you need additional aids to be able to carry out or follow the treatment (e.g. technology)?
  - What do you do in case of an emergency related to your disease?
- Please describe the type of telemedicine you are being treated with briefly!
  - How long have you been using this type of telemedical care?
  - How does your telemedical treatment work?
  - Which technical devices are used here?
  - What do you have to do in order to use this type of telemedicine?
  - Which documents do you need for using telemedicine?
  - How often do you have contact with the telemedical staff?
- What are your expectations of your telemedical treatment?
- How has your telemedical treatment affected your everyday life?
- What kind of relationship have you developed with the telemedical staff of your current treatment?
  - Is it important to you to build a relationship with the telemedical staff?
- How do you experience your telemedical treatment?

Examples:

- - Do you have confidence in your telemedical treatment?
  - Do you doubt your telemedical treatment?
  - Do you feel safer/ more insecure through your telemedical treatment?
  - Has the telemedical treatment changed your feeling of responsibility for your health?
  - Has the telemedical treatment changed your feeling of personal autonomy?
  - Has you perceived an extension or limitation of your possibilities by your telemedical treatment?
  - Has the telemedical treatment changed your awareness of your symptoms or condition?
- How do you evaluate your current telemedical treatment?
  - What are benefits of your current telemedical treatment?
  - What are disadvantages of your current telemedical treatment?
  - Are there any aspects of your current telemedical treatment that you would like to improve?
  - What is the main difference between the telemedical treatment and a standard treatment?
- Would you recommend your telemedical treatment to a friend?

1. **Quality of Life**

- What do you understand by the term *Quality of Life*?
- Please think freely: What does Quality of Life mean for you personally?
- Which areas of your life are particularly important for your Quality of Life?
- What role does health play in evaluating your Quality of Life?
- How has your disease affected your Quality of Life?
- What kind of impact does your current telemedical treatment have on your Quality of Life?
  - Which areas of your Quality of Life are affected?
  - Which of the stated areas improved? Which worsen?
- What would have to change in order to improve your Quality of Life?

Finally, is there anything you would like to add about your disease, treatment or Quality of Life?

*Interview guide: Professionals*

1. **Presentation of the participants (anonymous)**

- Profession, function, position, and responsibilities
- Current projects with reference to telemedicine
- Experience in/ use of telemedical applications
  - With which TM applications do you have concrete experience in care, development, assessment, etc.?
- Target group: Patients vs. users (diagnoses/ condition & socio-demographic data)
- Do you have a telemedicine-specific qualification/ training?
- Do you have care experience exclusively with reference to telemedical methods?

Agreement on which telemedical applications questions/ answers refer to. If they have a specific reference (answers can also refer to TM applications in general) - mark accordingly.

1. **Description of telemedical context the professional is working in**

- Please describe your current telemedical working environment!

If applicable:

- - How has the daily treatment routine of professionals changed since the use of telemedical care?
  - What are the bureaucratic procedures/ particularities (for professionals vs. patients/ users)?
  - Are there any additional costs for patients/ users?
  - In which contexts is the telemedical applications used (developed/ tested)
    (project/ insurance service/ private)?
  - Do you ever have face-to-face contact with patients/ users?
  - How are patients/ users informed about your telemedical services
    (or how should they be informed?)?
  - What experience have you gained so far with the use of telemedical methods?
- What are you aiming at with the use of telemedical methods in general
  (or with a selected TM method in particular) with regard to clinical, patient-side,
   economic, or other criteria?
- Which aspects of telemedical care should improve the quality of patient care?
- What kind of expectations do patients/ users express about telemedicine in the run-up to care/ application?
- What kind of concerns do patients/ users express about telemedicine in the run-up to care/ application?
- Do patients/ users have the opportunity to express their experiences with telemedicine during or after care? If so, what content is discussed?
- What do you think/ know about how patients/ users experience the use of technical devices in the context of telemedical care?
- How has the everyday life of your patients/ users changed through the use of telemedicine?
- In what form/ to what extent do (or can/ should) patients/ users using the telemedical application become active?
- In which patients/ users could you observe the effectiveness of telemedicine - and in which not?
- How has the quality of patient care changed since the use of telemedical methods?
- What do you know about patients/ users feeling more/less a) secured, b) supervised, c) supported through the use of the telemedical application?
- Can you identify changes in patients/ users with TM compared to patients/ users without TM?
- How do you evaluate the use of your telemedical application so far?
- What changes/ improvements in the field of telemedicine would you like to see?

1. **Quality of Life and Telemedicine**

- What do you understand by the term *Quality of Life* from a personal perspective?
- What do you understand by the term *Quality of Life* from your professional perspective?
- What do you think your patients/ users understand by the term *Quality of Life?*
- In your eyes, which areas of Quality of Life are affected in your patients/ users due to their condition?
- In your opinion, what should ideally be done to improve Quality of Life of your patients/users?
- How does your telemedical application affect the Quality of Life of your patients/ users?
- Which impact does/ could telemedicine have in general on the Quality of Life of patients with chronic conditions (physical and/ or mental)?
- Are there specific aspects of Quality of Life that in your opinion are specifically influenced by telemedical care in general (or a selected telemedical application in particular)?
- In addition to the aspects you have mentioned, are there any other patient-reported outcomes or experiences beyond the above-mentioned that are affected by telemedicine?

Finally, is there anything you would like to add?
