# Supplementary material for: Same same-but different: using qualitative studies to inform concept elicitation for quality of life assessment in telemedical care: a request for an extended working model
Source: Health Qual Life Outcomes. 2021 Jul 5;19:175. doi: 10.1186/s12955-021-01807-8 (PMC8256487; doi:10.1186/s12955-021-01807-8)
Supplement: Supplementary file 3 — Additional file 3. Supplementary C: A quantitative summary of the data evaluation. [file 12955_2021_1807_MOESM3_ESM.docx]

A quantitative summary of the data evaluation

The results are based on 6.987 codings in transcribed material from audio recordings of 15 focus groups and 63 single interviews: A total of 2.770 codes were assigned within the interviews and focus groups with patients (note that individual statements could be assigned to multiple categories). The code frequency in focus groups varied between 108 and 237 codes, while the number of codes in individual interviews ranged between 23 and 94 codes. "QoL" (730 codes) and "experiences with (TM) care" (2.040 codes) were deductively determined as main categories. Within the main category “QoL”, we generated two subcategories to separate the answers from patients with mental illnesses (48 subcategories with 386 codes) from patients with chronic physical diseases (46 subcategories with 344 codes). Among the chronically ill participants, the subcategories of the “QoL” main category with the most codes were "mobility" (45 codes) and "independent coping with own daily life" (25 codes), whereas "social contacts" (29 codes) and "social isolation" (22 codes) were most frequently mentioned in relation to QoL among participants with mental illnesses. The most frequently coded subcategory within the main category "experiences with (TM) care" were "safety experience" (125 codes), “perceived control” (84 codes) and “emergency aid” (71 codes).

Within interviews and focus groups with professionals, a total of 4.217 codes were assigned. The code frequency in focus groups varied between 100 and 229 codes, while the number of codes in individual interviews ranged between 73 and 155 codes. “QoL” (91 subcategories with 460 codes), "patient-related experiences with (TM) care" (96 subcategories with 2.547 codes) and "healthcare-professionals’ experiences with (TM) care" (44 subcategories with 1.213 codes) were deductively determined as main categories. "patient activation/empowerment" (37 codes), "knowledge and skills transfer" (27 codes), and "safety experience" (21 codes) were the subcategories most frequently coded under the main category of "QoL." Within the main category "patient-related experiences with (TM) care", “role of technology” (106 codes) and “empowerment/activation” (97codes) were most frequently assigned.
